# Eligibility criteria *vs*. need for pre-exposure prophylaxis: a reappraisal among men who have sex with men in Amsterdam, the Netherlands

**DOI:** 10.1017/S0950268822001741

**Published:** 2022-11-08

**Authors:** Feline de la Court, Anders Boyd, Udi Davidovich, Elske Hoornenborg, Maarten F. Schim Van Der Loeff, Henry J. C. De Vries, Daphne A. Van Wees, Birgit H. B. Van Benthem, Maria Xiridou, Amy Matser, Maria Prins

**Affiliations:** 1 Department of Infectious Diseases, Public Health Service of Amsterdam, Amsterdam, the Netherlands; 2 Stichting HIV Monitoring, Amsterdam, the Netherlands; 3 Department of Social Psychology, University of Amsterdam, Amsterdam, the Netherlands; 4 Department of Infectious Diseases, Amsterdam Institute for Infection & Immunity (AII), Amsterdam UMC, University of Amsterdam, Amsterdam, the Netherlands; 5 Department of Dermatology, Amsterdam UMC, University of Amsterdam, Amsterdam, the Netherlands; 6 Centre for Infectious Disease Control, National Institute for Public Health and the Environment (RIVM), Bilthoven, the Netherlands

**Keywords:** Chemsex, HIV/AIDS, men who have sex with men (MSM), pre-exposure prophylaxis (PrEP), sexually transmitted infections (STIs)

## Abstract

To reappraise pre-exposure prophylaxis (PrEP) eligibility criteria towards the men who have sex with men (MSM) with highest HIV-risk, we assessed PrEP need (i.e. HIV-risk) using Amsterdam Cohort Studies data from 2011–2017 for all non-PrEP using MSM. Outcomes were incident HIV-infection and newly-diagnosed anal STI. Determinants were current PrEP eligibility criteria (anal STI and condomless sex (CAS)) and additional determinants (age, education, group sex, alcohol use during sex and chemsex). We used targeted maximum likelihood estimation (TMLE) to estimate the relative risk (RR) and 95% confidence intervals (CI) of determinants on outcomes, and calculated population attributable fractions (PAFs) with 95% CI using RRs from TMLE. Among 810 included MSM, 22 HIV-infections and 436 anal STIs (*n* = 229) were diagnosed during follow-up. Chemsex (RR = 5.8 (95% CI 2.0–17.0); PAF = 55.3% (95% CI 43.3–83.4)), CAS with a casual partner (RR = 3.3 (95% CI 1.3–8.7); PAF = 38.0% (95% CI 18.3–93.6)) and anal STI (RR = 5.3 (95% CI 1.7–16.7); PAF = 22.0 (95% CI −16.8 to 100.0)) were significantly (*P* < 0.05) associated with and had highest attributable risk fractions for HIV. Chemsex (RR = 2.0 (95% CI 1.6–2.4); PAF = 19.5 (95% CI 10.6–30.6)) and CAS with a casual partner (RR = 2.5 (95% CI 2.0–3.0); PAF = 28.0 (95% CI 21.0–36.4)) were also significantly associated with anal STI, as was younger age (16–34/≥35; RR = 1.7 (95% CI 1.4–2.1); PAF = 15.5 (95% CI 6.4–27.6)) and group sex (RR = 1.3 (95% CI 1.1–1.6); PAF = 9.0 (95% CI −2.3 to 23.7)). Chemsex should be an additional PrEP eligibility criterion.

## Introduction

Pre-exposure prophylaxis (PrEP) is a highly effective HIV prevention strategy. PrEP uptake in high-income countries is relatively high among men who have sex with men (MSM) compared to other key populations [1]. Previous research from the Netherlands has shown that more than half of MSM at increased HIV risk report a high intention for PrEP use [2, 3]. Yet, while an estimated 16 730 MSM were at high risk for HIV in the Netherlands in 2019 [2, 3], only 5346 MSM were on PrEP through the national PrEP pilot in 2020 [4]. Despite increasing the national pilot capacity from 6500 to 8500 in 2021 and PrEP being available via general practitioners, the need for PrEP remains insufficiently met with the currently restricted provision capacity [3]. As PrEP access may remain restricted in the Netherlands and other regions with similar epidemics [1], a more in-depth understanding is needed of which criteria may be useful to allocate PrEP to those at the highest risk for HIV.

In the Netherlands, the current PrEP eligibility criteria pertain to MSM or transgender people who have had (1) insertive and/or receptive anal sex without a condom with a male partner with unknown HIV status, or with a known HIV-positive partner with a detectable viral load, (2) an anal sexually transmitted infection (STI), (3) syphilis or (4) used post-exposure prophylaxis (PEP); all reported or diagnosed in the preceding 6 months [5]. These clinical criteria were largely selected based on previous research denoting probable modes of HIV transmission and established HIV risk factors. Nevertheless, we lack understanding on how these criteria, along with potential alternative risk factors, contribute to HIV risk. Such insights could help provide the basis for more concrete decisions on whom should be prioritised for PrEP provision.

Given that HIV incidence has been greatly reduced in settings with universal treatment as prevention [6], any analysis examining the determinants of HIV-infection in settings, such as the Netherlands, and most other high-income countries will be limited by the low numbers of new infections and wide uncertainty around the parameter estimates modelling these determinants (i.e. insufficient power). As previous STIs are associated with HIV acquisition [7], inference on PrEP eligibility could also be made by studying determinants linked to the more common outcome, anal STI. Furthermore, previous studies examining the determinants of HIV and STI acquisition have relied on conventional methods in which the reasons for having a given risk-factor (i.e. risk-factor mechanism) and the outcome as predicted from the risk-factor and all other covariates (i.e. outcome mechanism) are susceptible to inappropriate modelling (i.e. model misspecification) [8, 9]. These techniques are also prone to overly large variance estimations, leading to statistical inefficiency, and can produce biased estimates when using a model containing subgroups with rare combinations of determinants, thereby violating the positivity assumption [8, 9].

In this study, we aimed to determine the characteristics of MSM most at risk for HIV-infection or anal STI (as a proxy for HIV risk) as a means to identify those who could benefit most from PrEP using data from the Amsterdam Cohort Study (ACS). To this end, we assessed the association between criteria and newly-diagnosed HIV or anal STI using targeted maximum likelihood estimation (TMLE), a method designed to accommodate the problems arising from conventional statistical techniques mentioned above [8, 9], while using parameters from these estimates to calculate the population attributable fractions (PAF) of each criterion. We intended to use this information to reappraise the relevance of current, as well as potential additional, criteria for PrEP eligibility.

## Methods

### Study design and population

The ACS is an open, ongoing prospective cohort study initiated in 1984 at the Public Health Service of Amsterdam (GGD). Between 2011 and 2017, recruitment took place among men who had sex with at least one man in the previous 6 months and who either lived in Amsterdam or had regular male sexual contacts or MSM-related activities in Amsterdam. Age at inclusion was limited to 30 years during several time periods to reduce age-induced bias resulting from a uniformly ageing cohort. Recruitment took place via convenience sampling, including outreach activities and online advertisements on gay dating apps, and via chain referral sampling.

We used data from 2011–2017 for HIV-negative participants and those with an incident HIV-infection during this follow-up period. This restricts data to a timeframe during which perceived HIV risk was relatively high among MSM in Amsterdam [10] and includes data from a period before affordable PrEP became available (2018) and the national PrEP pilot was implemented (2019).

The ACS was approved by the Medical Ethics Committee of the Amsterdam University Medical Centre of the University of Amsterdam, the Netherlands (MEC-07/182). Participation is voluntary and written informed consent was provided by participants at enrolment.

### Data collection

At each biannual ACS visit, participants completed a self-administered questionnaire and were tested for HIV and bacterial STI. Questionnaire data pertained to sociodemographic characteristics, sexual behaviour, psychosocial risk determinants and substance use. Testing was performed for HIV antigens and antibodies, and for bacterial STI including *Treponema pallidum* (syphilis), *Chlamydia trachomatis* and *Neisseria gonorrhoeae.* Extra visits for additional HIV and/or STI testing were possible between study visits in case of STI-related symptoms, partner notification for an STI or to participant's discretion. Analyses were restricted to include only study visits for which the questionnaire was completed and HIV and STI test results were available.

### Study variables

We used data on the following dichotomous variables, which relate to the current Dutch PrEP eligibility criteria [5]: anal STI (chlamydia and/or gonorrhoea), condomless anal sex with a steady partner, and condomless anal sex with a casual partner; all referring to the 6 months prior to the study visit. Because few participants reported PEP use (102/6661 visits with reported PEP use; 1.5%) or were diagnosed with syphilis (76 visits with diagnosed syphilis in previous 6 months; 1.0%), these two PrEP eligibility criteria could not be analysed. We additionally censored participants in the analysis when they began PrEP use or became HIV-positive during follow-up.

We additionally included data on sociodemographic characteristics (age 16–34 *vs*. ≥35, defined as ‘younger’ and ‘older’, respectively; and education level, college/university degree or not, defined as ‘higher’ and ‘lower’, respectively) and other sexual behaviour variables (group sex, yes/no; alcohol use during or prior to sex, yes/no; and chemsex, yes/no, defined as gamma-Hydroxybutyrate (GHB), gamma-butyrolactone (GLB) mephedrone, methamphetamine, ketamine, amphetamine, cocaine or 3,4-Methyl-enedioxy-methamphetamine (MDMA) use during or prior to sex [11]; all occurring in the 6 months prior to the study visit).

### Statistical analyses

The two outcomes in this study were (1) incident HIV-infection, and (2) any newly-diagnosed anal STI infection (in the past 6 months) as a proxy for HIV-infection [12]. We first described population characteristics between 2011 and 2017 for all included participants at baseline (i.e. first visit in the study period), for those who became HIV-positive (at the visit of their positive test), and for those who tested positive for anal STI (at the visit of their first anal STI diagnosis).

Secondly, we used TMLE to estimate the target parameter of a relative risk (RR) along with its 95% confidence intervals (CI) from binary exposure and outcome variables [8]. Briefly, this method makes an initial estimate of the conditional mean probability of the outcome given a specific determinant and other covariates (outcome regression). The probability of belonging to a given level of the determinant is then modelled as a function of the other covariates (propensity score regression). The estimate of the outcome regression is updated using information from the propensity score regression in an iterative manner until convergence is reached. The target parameter is then estimated from outputs of the outcome and propensity score regression and variance estimators obtained based on an efficient influence curve. To avoid further model misspecification, both the outcome and propensity score regression are optimised using an ensemble of machine learning techniques, referred to as a ‘super learner’ [9]. The weighted combination of predictions by which the cross-validated mean square error is minimised is selected through the super learner. For this study, a target parameter was calculated for each determinant, while accounting for all determinants listed in the ‘Study variables’ except those used as an outcome.

Estimates were constructed using the ‘tmle’ and ‘SuperLearner’ packages in R. The ensembles included the following: generalised linear models (with and without interactions), generalised additive models, regression trees, random forests (minimum node sizes of 50, 100, 150 and 200 individuals), extreme gradient boosting (with the same node specifications as in the random forests with combinations of shrinkage parameters at 0.001, 0.01 and 0.1), and elastic net regression (alpha at 0, 0.2, 0.4, 0.6, 0.8 and 1). Observations were treated as independent and identically distributed, meaning that standard errors were not corrected for repeated measures within participants. Observations with missing values were excluded from the analyses.

Thirdly, PAFs were calculated from the RR obtained from TLME and presented as percentages, specifically for determinants of anal STI and HIV that indicated an increased risk (RR > 1) [13]. PAF estimates indicate the proportion of HIV or anal STI cases that are attributable to exposure to a determinant, assuming causality. We used the formula PAF *=* *P*_e_ (RR *−* 1)*/*(*P*_e_(RR − 1) + 1), where *P*_e_ is the prevalence of exposed individuals. The ‘pifpaf’ package in R was employed to estimate PAFs along with the corresponding 95% CI using a bootstrap approximation with 2000 replicates.

All RR and PAF calculations are adjusted as part of the TMLE procedure. Significance was defined at *P* < 0.05 and analysis was performed using R (v3.6.3, Vienna, Austria).

## Results

### Study population

Between 2011 and 2017, 9969 visits occurred in 969 participants (median visits per participant = 11; IQR = 6–14). We excluded 3308 study visits of 159 participants. Of these visits, 601 were excluded due to missing outcome data, 2449 due to missing questionnaire data, 163 due to reported PrEP use, and for the 22 participants who became HIV-positive during follow-up, the 95 follow-up visits that took place after HIV diagnosis. In total, 810 participants were included in analyses, contributing 6661 visits (median visits per participant = 9; IQR = 5–12). Supplementary Table S1 shows the annual number of visits, number of participants, distribution of age and number of visits (with HIV/STI testing) per participant from 2011 and 2017.

During follow-up, there were 22 incident HIV-infections. An anal STI was diagnosed in 6.6% of visits, amounting to 436 anal STI diagnoses in 229 participants, among whom the median number of anal STI diagnoses per participant was 2 (IQR = 1–2). Table 1 describes the population characteristics at baseline and at diagnosis (for those who became HIV-positive and who tested positive for an anal STI).

**Table 1.** Characteristics of HIV-negative participants of the ACS between 2011 and 2017 at first visit (baseline), at HIV-infection and at first anal STI diagnosis during follow-up

	Total population (At baseline)	At HIV-infection (During follow-up)	At first anal STI (During follow-up)
	
	*n* = 810^a^	*n* = 22^b^	*n* = 229^c^
Age (years)	36.1 (29.6–43.0)	37.5 (29.0–41.4)	37.5 (29.0–41.4)
≥35	458 (56.5%)	13 (59.1%)	127 (58.3%)
16–34	352 (43.5%)	9 (40.9%)	91 (41.7%)
Education level			
Low (no college/university)	187 (23.1%)	5 (22.7%)	55 (25.2%)
High (college/university)	622 (76.9%)	17 (77.3%)	163 (74.8%)
Condomless anal sex with a casual partner			
No	599 (75.3%)	7 (31.8%)	110 (50.7%)
Yes	197 (24.8%)	15 (68.2%)	107 (49.3%)
Condomless anal sex with a steady partner			
No	492 (61.9%)	16 (72.7%)	144 (67.3%)
Yes	303 (38.1%)	6 (27.3%)	70 (32.7%)
Chemsex			
No	562 (72.0%)	5 (23.8%)	123 (57.5%)
Yes	219 (28.0%)	16 (76.2%)	91 (42.52%)
Alcohol during sex			
No	333 (42.5%)	10 (45.5%)	83 (38.8%)
Yes	451 (57.5%)	12 (54.6%)	131 (61.2%)
Group sex			
No	522 (65.5%)	7 (31.8%)	113 (52.1%)
Yes	275 (34.5%)	15 (68.2%)	104 (47.9%)

Data are presented as medians (IQR) or *n* (percentage); baseline refers to the first visit per participant between 2011 and 2017; ‘at first anal STI’ refers to the first visit during which an anal STI was diagnosed between 2011 and 2017; ‘at HIV-infection’ refers to the visit during which HIV was diagnosed. Chemsex is defined as GBL, GHB, mephedrone, methamphetamine, ketamine, amphetamine, cocaine or XTC use during or prior to sex. All variables refer to the 6 months prior to the follow-up visits.

a Number of participants with missing data at baseline: education level 1, condomless anal sex with a casual partner 14, condomless anal sex with a steady partner 15, chemsex 29, alcohol during sex 26, group sex 13.

b Number of participants with missing data at HIV infection: chemsex 1.

c Number of participants with missing data at first anal STI: age 11, education level 11, condomless anal sex with a casual partner 12, condomless anal sex with a steady partner 15, chemsex 25, alcohol during sex 15, group sex 12.

### Individuals at risk of HIV and anal STI

Table 2 reports the RR estimates from TLME with 95% CI per determinant, alongside the number of visits during which the determinant was reported for both the total population and stratified for HIV outcome. The risk of becoming HIV-positive was significantly higher for those who had condomless anal sex with a casual partner (*P* = 0.02), engaged in chemsex (*P* = 0.001), or had an anal STI diagnosis in the previous 6 months (*P* = 0.004).

**Table 2.** Determinants of incident HIV-infections among participants of the ACS between 2011 and 2017 obtained with TMLE

	Number of study visits (%)			TMLE estimate	
	Total	HIV absent	HIV present	RR (95% CI)	*P*
Age (years)					
≥35	4968 (74.6%)	4955 (74.6%)	13 (59.1%)	Reference	
16–34	1693 (25.4%)	1684 (25.4%)	9 (40.9%)	1.5 (0.6–3.6)	0.41
Education level					
Low (no college/university)	1534 (23.0%)	1529 (23.0%)	5 (22.7%)	Reference	
High (college/university)	5126 (77.0%)	5109 (77.0%)	17 (77.3%)	1.3 (0.8–2.0)	0.27
Condomless anal sex with a casual partner					
No	4875 (73.5%)	4868 (73.7%)	7 (31.8%)	Reference	
Yes	1755 (26.5%)	1740 (26.3%)	15 (68.2%)	3.3 (1.3–8.7)	0.02
Condomless anal sex with a steady partner					
No	4069 (61.6%)	4053 (61.6%)	16 (72.7%)	Reference	
Yes	2533 (38.4%)	2527 (38.4%)	6 (27.3%)	0.5 (0.2–1.2)	0.11
Chemsex					
No	4831 (74.5%)	4826 (74.6%)	5 (23.8%)	Reference	
Yes	1657 (25.5%)	1641 (25.4%)	16 (76.2%)	5.8 (2.0–17.0)	0.001
Alcohol during sex					
No	2789 (42.9%)	2779 (42.9%)	10 (45.5%)	Reference	
Yes	3713 (57.1%)	3701 (57.1%)	12 (54.6%)	0.5 (0.2–1.1)	0.07
Group sex					
No	4549 (68.6%)	4542 (68.7%)	7 (31.8%)	Reference	
Yes	2087 (31.5%)	2072 (31.3%)	15 (68.2%)	2.2 (0.9–5.4)	0.10
Anal STI					
Negative	6225 (93.5%)	6212 (93.6%)	13 (59.1%)	Reference	
Positive	436 (6.6%)	427 (6.4%)	9 (40.9%)	5.3 (1.7–16.7)	0.004

For number of study visits: data are presented as *n* (percentages); for TMLE estimate: the target parameter is presented as RR (95% CI). Explanation of data: RR, risk ratio; 95% CI, 95% confidence interval; *P*, *P*-value (significance defined at *P*-value <0.05); TMLE, targeted maximum likelihood estimation. Chemsex is defined as GBL, GHB, mephedrone, methamphetamine, ketamine, amphetamine, cocaine or XTC use during or prior to sex. Anal STI means diagnosis with anal chlamydia and/or anal gonorrhoea in the 6 months prior to the follow-up visit. All variables refer to the 6 months prior to the follow-up visits.

As shown in Table 3, the risk of being diagnosed with an anal STI was significantly higher for those who had condomless anal sex with a casual partner (*P* < 0.0001), engaged in chemsex (*P* < 0.0001), group sex (*P* = 0.01) and among those aged 16–34 compared to 35 years or older (*P* < 0.0001). Those reporting any condomless anal sex with a steady partner (*P* = 0.01) and alcohol use during or prior to sex (*P* = 0.01) had a significantly lower risk of being diagnosed with an anal STI.

**Table 3.** Determinants of anal STI among HIV-negative participants of the ACS between 2011 and 2017 using TMLE

	Number of study visits (%)			TMLE estimate	
	Total	Anal STI absent	Anal STI present	RR (95% CI)	*P*
Age (years)					
≥35	4968 (74.6%)	4700 (75.5%)	268 (61.5%)	Reference	
16–34	1693 (25.4%)	1525 (24.5%)	168 (38.5%)	1.7 (1.4–2.1)	<0.0001
Education level					
Low (no college/university)	1534 (23.0%)	1416 (22.8%)	118 (27.1%)	Reference	
High (college/university)	5126 (77.0%)	4808 (77.3%)	318 (72.9%)	0.9 (0.7–1.1)	0.21
Condomless anal sex with a casual partner					
No	4875 (73.5%)	4671 (75.4%)	204 (47.0%)	Reference	
Yes	1755 (26.5%)	1525 (24.6%)	230 (53.0%)	2.5 (2.0–3.0)	<0.0001
Condomless anal sex with a steady partner					
No	4069 (61.6%)	3776 (61.2%)	293 (68.0%)	Reference	
Yes	2533 (38.4%)	2395 (38.8%)	138 (32.0%)	0.8 (0.6–1.0)	0.01
Chemsex					
No	4831 (74.5%)	5418 (89.2%)	314 (73.7%)	Reference	
Yes	1657 (25.5%)	655 (10.8%)	112 (26.3%)	2.0 (1.6–2.4)	<0.0001
Alcohol during sex					
No	2789 (42.9%)	2622 (43.2%)	167 (39.1%)	Reference	
Yes	3713 (57.1%)	3453 (56.8%)	260 (60.9%)	0.8 (0.7–1.0)	0.01
Group sex					
No	4549 (68.6%)	4310 (69.5%)	239 (54.9%)	Reference	
Yes	2087 (31.5%)	1891 (30.5%)	196 (45.1%)	1.3 (1.1–1.6)	0.01

For number of study visits: data are presented as *n* (percentages); for TMLE estimate: the target parameter is presented as RR (95% CI). Explanation of data: RR, risk ratio; 95% CI, 95% confidence interval; *P*, *P*-value (significance defined at *P*-value <0.05); TMLE, targeted maximum likelihood estimation. Chemsex is defined as GBL, GHB, mephedrone, methamphetamine, ketamine, amphetamine, cocaine or XTC use during or prior to sex. Anal STI means diagnosis with anal chlamydia and/or anal gonorrhoea in the 6 months prior to the follow-up visit. All variables refer to the 6 months prior to the follow-up visits.

### PAF of risk factors for HIV and anal STI

**Fig. 1.**
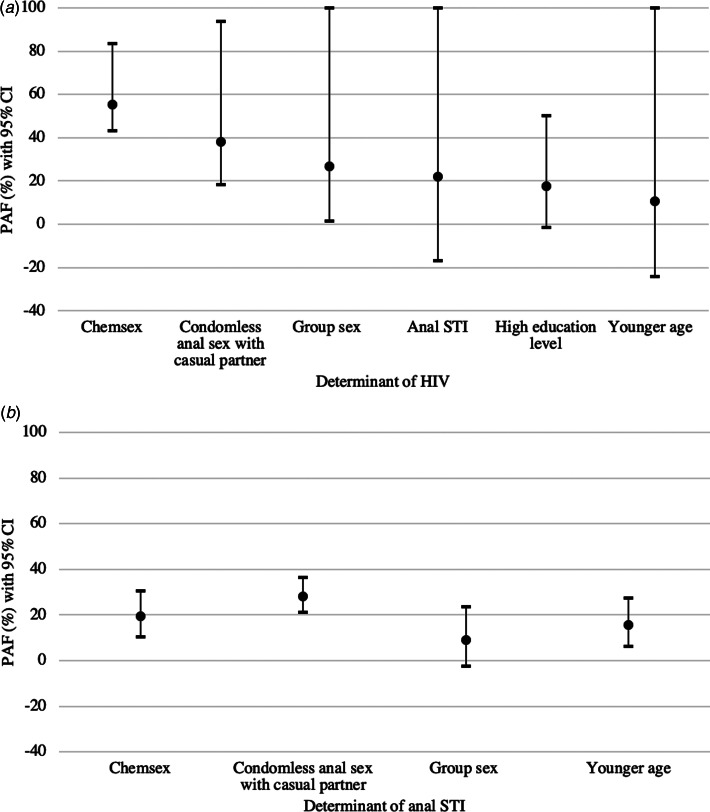
PAFs with 95% CI for determinants of (a) HIV infection, and (b) anal STI (chlamydia or gonorrhoea) among participants of the ACS between 2011 and 2017. PAFs were calculated from the RR obtained from TLME and presented as percentages with 95% CI, including only determinants of anal STI and HIV that indicated an increased risk (i.e. RR > 1). All included variables refer to the 6 months prior to the follow-up visits. Chemsex is defined as GBL, GHB, mephedrone, methamphetamine, ketamine, amphetamine, cocaine or XTC use during or prior to sex. Anal STI means diagnosis with anal chlamydia and/or anal gonorrhoea in the 6 months prior to, or at the follow-up visit. Younger age means aged 16–34 *vs*. ages 35 and above. High education level means having a college or university degree and is compared to low education level, meaning no college or university degree.

The PAF estimates with 95% CI are depicted in Fig. 1. Chemsex had the highest attributable risk fraction for HIV (PAF = 55.3%; 95% CI 43.3–83.4) followed by condomless anal sex with a casual partner (PAF = 38.0%; 95% CI 18.3–93.6), group sex (PAF = 26.7%; 95% CI 1.6–100.0), anal STI (PAF = 22.0; 95% CI −16.8 to 100.0), higher education level (PAF = 17.4%; 95% CI −1.3 to 50.3) and younger age (PAF = 10.7%; 95% CI −24.1 to 100.0). However, the lower limit of the PAF 95% CI for age, education level and anal STI were below 0%.

For anal STI, condomless anal sex with a casual partner had the highest attributable risk fraction (PAF = 28.0; 95% CI 21.0–36.4), followed by chemsex (PAF = 19.5; 95% CI 10.6–30.6), younger age (PAF = 15.5; 95% CI 6.4–27.6) and group sex (PAF = 9.0; 95% CI −2.3 to 23.7). The lower limit of the PAF 95% CI for group sex was below 0%. All PAF values with corresponding 95% CI are reported in Supplementary Table S2.

## Discussion

Using extensive longitudinal data from the ACS, we demonstrated that condomless anal sex with a casual partner, anal STI and chemsex in the past 6 months were associated with newly-diagnosed HIV among MSM, with considerably strong effect sizes and large PAFs for all three determinants. Including chemsex as an additional PrEP eligibility criterion among MSM could thus further tailor PrEP provision and improve HIV prevention outcomes. Chemsex and condomless anal sex with a casual partner were also associated with anal STI, as well as younger age and group sex. Given the strong relationship between HIV and anal STI, our findings implicate that these determinants are also important candidates for PrEP eligibility criteria. Our study provides valuable information to determine which additional characteristics can be used to identify those who can benefit most from PrEP use.

Condomless anal sex, especially with casual partners, has previously been established as the most prominent risk behaviour for HIV [14]. It is then unsurprising that condomless anal sex with casual partners is a criterion for PrEP eligibility across all major PrEP guidelines [15]. Moreover, our results show that chemsex was the most important determinant for HIV-infection, having both the highest RR from TMLE and highest PAF estimate. Although chemsex itself is not a transmission factor, MSM engaging in chemsex often exhibit other behaviours that increase their risk for HIV-infection [16], including prolonged sexual interactions, experiencing non-consensual sex, condomless anal sex with multiple partners and possibly injection drug use with potential risk of sharing injecting equipment [17].

Given that the association between chemsex and CAS has previously been demonstrated [18], it could be argued that adding chemsex to the PrEP use criteria would be redundant. However, the RR was much stronger for chemsex than CAS and considering that 80% of MSM engaging in chemsex also had CAS, they could also represent a subset of individuals reporting CAS at heightened risk of HIV. Coupled with the expected additional outreach and the examples set by the EACS guidelines [15] and countries like France [19] and England [20] who already include chemsex in their PrEP guidelines, the addition of chemsex to the PrEP eligibility criteria in the Netherlands, and in countries with similar epidemics, seems warranted.

Group sex and younger age were found to be significantly associated with anal STI, but not for HIV; likely due to the limited number of HIV-infections. Nonetheless, group sex and younger age did appear among the five highest attributable risk fractions for HIV based on their PAF values. Previous research has shown the association between these determinants and both STI and HIV infections [21]. Unprotected exposure to multiple partners during group sex is a plausible scenario, especially when also involving chemsex [22]. Moreover, younger MSM have been identified as a potentially vulnerable group for HIV-infection due to their relatively large sexual networks and relative unawareness of sexual behaviours associated with increased STI/HIV risk [23]. It could be helpful to additionally apply these determinants as co-indicators for PrEP eligibility.

From TMLE, we also found that condomless anal sex with a steady partner had a protective effect for both HIV and STI infection. This is likely attributable to the commonly-applied concept of ‘negotiated safety’ within steady partnerships, referring to sexual agreements on HIV testing, prevention and exclusivity within a relationship [24]. It is important to keep promoting the discussion of prevention strategies within steady relationships and evaluate lack thereof when considering PrEP eligibility [25].

Surprisingly, alcohol use during or prior to sex also showed a protective effect regarding HIV and STI outcomes, albeit not significantly. According to a study in Amsterdam, the fraction reporting condomless anal sex, number of sex partners and STI prevalence were all lower among MSM reporting alcohol but no drug use during or prior to sex, compared to those reporting drug use [26]. Because alcohol use is often associated with drug use and chemsex [27], the direct effect of alcohol use alone might be difficult to estimate.

Although PrEP should be made widely available, high demand and budgetary restrictions require targeted and rapid rollout of PrEP towards those with the highest HIV risk. Tailoring the eligibility criteria of PrEP further does bare certain considerations. Use of the current PrEP criteria has been modelled to be cost-effective in the Netherlands [28], especially among MSM at high risk for HIV [29]. Costs may increase disproportionately in relation to the benefits if PrEP access is specified to subgroups of MSM with lower risk of acquiring HIV than those currently eligible, depending on the size of these subgroups. Furthermore, simply altering access to PrEP based on a given set of criteria might not necessarily lead to increased uptake in certain key populations. Insight is therefore needed into what characterises eligible non-users and to what extent eligibility recognition and increasing PrEP awareness (i.e. demand creation) can be altered to tailor to those who would benefit most from PrEP use. Further research should combine qualitative and quantitative methods to gain a deeper understanding of how PrEP implementation can be improved, specifically regarding barriers and missed opportunities, and for which groups PrEP implementation needs to be tailored.

Study limitations include, first, the limited generalisability of the ACS population, as it consists of mainly Dutch, middle aged and highly educated MSM. There is thus an underrepresentation of individuals who may be at a higher HIV risk, such as younger individuals and migrant groups among whom PrEP uptake is notably lower [4]. Secondly, because all analyses were performed at the visit level, we could not account for repeated measurements. It is unclear how appropriate variances estimates are in the present study considering that PrEP eligibility is known to vary within individuals [30], and an individual's behaviour can vary widely between visits. Thirdly, our study was also restricted by the limited number of HIV-infections, which decreased the power to detect determinants of HIV acquisition and increased the uncertainty around the PAF estimates for HIV-infection. To mitigate this limitation, we examined determinants and PAF for anal STI, which was strongly associated with incident HIV infection in our study and has been suggested as a proxy for HIV-risk [12]. Fourthly, although the WHO recommends that PrEP guidelines consider the higher HIV risk that comes with having CAS with multiple partners [31], we could not analyse the number of CAS partners as the extensiveness of our questionnaire restricted us to only ask questions on the total number of sexual partners, regardless of condom use. Finally, two PrEP criteria, PEP use and syphilis, were excluded from our analyses because they rarely occurred among participants, despite being important HIV risk factors commonly included in PrEP guidelines [15].

An important strength of this study is that the ACS cohort provides unique and detailed longitudinal data for both HIV and anal STI outcomes. Within the ACS population, there are MSM engaging in a wide range of levels of risks associated HIV and anal STI, which makes it possible to establish determinants and allows us to compare results from HIV and anal STI outcomes. Furthermore, the use of TMLE allowed for an estimation of associated effects with minimal bias, decreased risk in model misspecification, and requires weaker assumptions compared to conventional regression techniques [9]. These methods, in addition to the use of anal STI as HIV proxy, addresses some of the methodological limitations of previous HIV studies with low HIV incidence, allowing for more informative conclusions. Lastly, PAF provides a valuable addition to TMLE.

In conclusion, MSM in Amsterdam, the Netherlands who would benefit most from PrEP are those having condomless anal sex with a casual partner, anal STI and chemsex in the past 6 months. We therefore provide a much-needed understanding of which added criteria are useful to allocate PrEP to those at the highest risk of HIV. We suggest that chemsex should be added to the eligibility criteria in the Dutch guidelines and guidelines from regions with similar epidemics. Furthermore, it may be beneficial for HIV risk reduction if priority for PrEP is given to those who are younger or those engaging in group sex. We emphasise the need for further research to elaborate on PrEP eligibility and the costs and benefits thereof for optimal PrEP rollout.

## Data Availability

The data used in this study are available from the corresponding author (F. d. l. C.) upon reasonable request.

## Supplementary material

For supplementary material accompanying this paper visit http://doi.org/10.1017/S0950268822001741. click here to view supplementary material
