# Supplementary material for: Eligibility criteria vs. need for pre-exposure prophylaxis: a reappraisal among men who have sex with men in Amsterdam, the Netherlands
Source: Epidemiol Infect. 2022 Nov 8;150:e190. doi: 10.1017/S0950268822001741 (PMC9987018; doi:10.1017/S0950268822001741)
Supplement: Supplementary file 1 [file hygsup.zip › S0950268822001741sup001.docx]

**Supplementary material**

**Supplementary table 1.** *Annual number of visits, participants, age distribution and number of visits (with HIV/STI testing) per participant in the Amsterdam Cohort Studies (ACS) between 2011 and 2017*

| **Year** | **Number of visits** | **Number of participants** | **Age (years)** | **Number of visits* per participant** | |
| --- | --- | --- | --- | --- | --- |
|  |  |  | Median [IQR] | Median [IQR] | Mean (SD) |
| 2011 | 856 | 471 | 38.2 [33.9-43.1] | 2 [2-2] | 1.8 (0.5) |
| 2012 | 848 | 487 | 39.1 [34.6-44.2] | 2 [1-2] | 1.7 (0.4) |
| 2013 | 942 | 530 | 40.3 [35.4-45.2] | 2 [2-2] | 1.8 (0.4) |
| 2014 | 1076 | 610 | 40.4 [35.3-46.0] | 2 [1.25-2] | 1.8 (0.5) |
| 2015 | 1019 | 634 | 41.0 [35.0-47.4] | 2 [1-2] | 1.6 (0.5) |
| 2016 | 960 | 574 | 41.6 [35.0-47.9] | 2 [1-2] | 1.7 (0.5) |
| 2017 | 960 | 566 | 42.6 [35.3-48.8] | 2 [1-2] | 1.7 (0.5) |

Explanation of data: IQR = interquartile range; SD = standard deviation. Note: * = at all included visits both STI and HIV testing took place.

**Supplementary table 2.** *PAF for determinants of anal STI and HIV among participants of the Amsterdam Cohort Studies (ACS) between 2011 and 2017*

|  |  | **HIV** | **Anal STI** |
| --- | --- | --- | --- |
|  |  | PAF in percentage (95% CI) | PAF in percentage (95% CI) |
| Age *16-34* years | | 10.7 (-24.1-100.0) | 15.5 (6.4-27.6) |
| *High (college/university*) education level | | 17.4 (-1.3-50.3) | * |
| Condomless anal sex with a casual partner | | 38.0 (18.3-93.6) | 28.0 (21.0-36.4) |
| Condomless anal sex with a steady partner | | * | * |
| Chemsex | | 55.3 (43.3-83.4) | 19.5 (10.6-30.6) |
| Alcohol during sex | | * | * |
| Group sex | | 26.7 (1.6-100.0) | 9.0 (-2.3-23.7) |
| Anal STI diagnosis | | 22.0 (-16.8-100.0) | - |

Explanation of data: PAF = population attributable fraction; 95% CI = 95% confidence interval. Anal STI means diagnosis with anal chlamydia and/or anal gonorrhoea in the 6 months prior to the follow-up visit. Chemsex is defined as GBL, GHB, mephedrone, methamphetamine, ketamine, amphetamine, cocaine, or XTC use during or prior to sex. All variables refer to the six months prior to the follow-up visits. Note: *= not calculated because RR was less than 1.
