# Supplementary material for: Eligibility criteria vs. need for pre-exposure prophylaxis: a reappraisal among men who have sex with men in Amsterdam, the Netherlands
Source: Epidemiol Infect. 2022 Nov 8;150:e190. doi: 10.1017/S0950268822001741 (PMC9987018; doi:10.1017/S0950268822001741)
Supplement: Supplementary file 1 [file hygsup.zip › S0950268822001741sup002.docx]

**Tables and figures**

**Table 1.** *Characteristics of HIV-negative participants of the Amsterdam Cohort Studies (ACS) between 2011-2017 at first visit (baseline), at HIV-infection and at first anal STI diagnosis during follow-up*

|  | | **Total population**  (At baseline) | **At HIV-infection**  (During follow-up) | **At first anal STI**  (During follow-up) |
| --- | --- | --- | --- | --- |
|  | | n=810^*^ | n=22^**^ | n=229^***^ |
| Age (years) | | 36.1 [29.6-43.0] | 37.5 [29.0-41.4] | 37.5 [29.0-41.4] |
|  | ≥35 | 458 (56.5%) | 13 (59.1%) | 127 (58.3%) |
|  | *16-34* | 352 (43.5%) | 9 (40.9%) | 91 (41.7%) |
| Education level | |  |  |  |
|  | *Low (no college/university)* | 187 (23.1%) | 5 (22.7%) | 55 (25.2%) |
|  | *High (college/university)* | 622 (76.9%) | 17 (77.3%) | 163 (74.8%) |
| Condomless anal sex with a casual partner | |  |  |  |
|  | *No* | 599 (75.3%) | 7 (31.8%) | 110 (50.7%) |
|  | *Yes* | 197 (24.8%) | 15 (68.2%) | 107 (49.3%) |
| Condomless anal sex with a steady partner | |  |  |  |
|  | *No* | 492 (61.9%) | 16 (72.7%) | 144 (67.3%) |
|  | *Yes* | 303 (38.1%) | 6 (27.3%) | 70 (32.7%) |
| Chemsex | |  |  |  |
|  | *No* | 562 (72.0%) | 5 (23.8%) | 123 (57.5%) |
|  | *Yes* | 219 (28.0%) | 16 (76.2%) | 91 (42.52%) |
| Alcohol during sex | |  |  |  |
|  | *No* | 333 (42.5%) | 10 (45.5%) | 83 (38.8%) |
|  | *Yes* | 451 (57.5%) | 12 (54.6%) | 131 (61.2%) |
| Group sex | |  |  |  |
|  | *No* | 522 (65.5%) | 7 (31.8%) | 113 (52.1%) |
|  | *Yes* | 275 (34.5%) | 15 (68.2%) | 104 (47.9%) |

Data are presented as medians [IQR] or n (percentage); baseline refers to the first visit per participant between 2011 and 2017; “at first anal STI” refers to the first visit during which an anal STI was diagnosed between 2011-2017; “at HIV-infection” refers to the visit during which HIV was diagnosed. Chemsex is defined as GBL, GHB, mephedrone, methamphetamine, ketamine, amphetamine, cocaine, or XTC use during or prior to sex. All variables refer to the six months prior to the follow-up visits.

*Number of participants with missing data at baseline: education level 1, condomless anal sex with a casual partner 14, condomless anal sex with a steady partner 15, chemsex 29, alcohol during sex 26, group sex 13.

**Number of participants with missing data at HIV infection: chemsex 1.

**Number of participants with missing data at first anal STI: age 11, education level 11, condomless anal sex with a casual partner 12, condomless anal sex with a steady partner 15, chemsex 25, alcohol during sex 15, group sex 12.

**Table 2.** *Determinants of incident HIV-infections among participants of the Amsterdam Cohort Studies (ACS) between 2011-2017 obtained with targeted maximum likelihood estimation*

|  |  | **Number of study visits (%)** | | | **TMLE estimate** | |
| --- | --- | --- | --- | --- | --- | --- |
|  |  | Total | HIV absent | HIV present | RR (95% CI) | *p* |
| Age (years) | |  |  |  |  |  |
|  | ≥35 | 4,968 (74.6%) | 4,955 (74.6%) | 13 (59.1%) | Reference |  |
|  | *16-34* | 1,693 (25.4%) | 1,684 (25.4%) | 9 (40.9%) | 1.5 (0.6-3.6) | 0.41 |
| Education level | |  |  |  |  |  |
|  | *Low (no college/university)* | 1,534 (23.0%) | 1,529 (23.0%) | 5 (22.7%) | Reference |  |
|  | *High (college/university)* | 5,126 (77.0%) | 5,109 (77.0%) | 17 (77.3%) | 1.3 (0.8-2.0) | 0.27 |
| Condomless anal sex with a casual partner | |  |  |  |  |  |
|  | *No* | 4,875 (73.5%) | 4,868 (73.7%) | 7 (31.8%) | Reference |  |
|  | *Yes* | 1,755 (26.5%) | 1,740 (26.3%) | 15 (68.2%) | 3.3 (1.3-8.7) | 0.02 |
| Condomless anal sex with a steady partner | |  |  |  |  |  |
|  | *No* | 4,069 (61.6%) | 4,053 (61.6%) | 16 (72.7%) | Reference |  |
|  | *Yes* | 2,533 (38.4%) | 2,527 (38.4%) | 6 (27.3%) | 0.5 (0.2-1.2) | 0.11 |
| Chemsex | |  |  |  |  |  |
|  | *No* | 4,831 (74.5%) | 4,826 (74.6%) | 5 (23.8%) | Reference |  |
|  | *Yes* | 1,657 (25.5%) | 1,641 (25.4%) | 16 (76.2%) | 5.8 (2.0-17.0) | 0.001 |
| Alcohol during sex | |  |  |  |  |  |
|  | *No* | 2,789 (42.9%) | 2,779 (42.9%) | 10 (45.5%) | Reference |  |
|  | *Yes* | 3,713 (57.1%) | 3,701 (57.1%) | 12 (54.6%) | 0.5 (0.2-1.1) | 0.07 |
| Group sex | |  |  |  |  |  |
|  | *No* | 4,549 (68.6%) | 4,542 (68.7%) | 7 (31.8%) | Reference |  |
|  | *Yes* | 2,087 (31.5%) | 2,072 (31.3%) | 15 (68.2%) | 2.2 (0.9-5.4) | 0.10 |
| Anal STI | |  |  |  |  |  |
|  | *Negative* | 6,225 (93.5%) | 6,212 (93.6%) | 13 (59.1%) | Reference |  |
|  | *Positive* | 436 (6.6%) | 427 (6.4%) | 9 (40.9%) | 5.3 (1.7-16.7) | 0.004 |

For number of study visits: data are presented as n (percentages); for TMLE estimate: the target parameter is presented as RR (95% CI). Explanation of data: RR = risk ratio; 95% CI = 95% confidence interval; p = p-value (significance defined at p-value <0.05); TMLE = targeted maximum likelihood estimation. Chemsex is defined as GBL, GHB, mephedrone, methamphetamine, ketamine, amphetamine, cocaine, or XTC use during or prior to sex. Anal STI means diagnosis with anal chlamydia and/or anal gonorrhoea in the 6 months prior to the follow-up visit. All variables refer to the six months prior to the follow-up visits.

**Table 3.** *Determinants of anal STI among HIV-negative participants of the Amsterdam Cohort Studies (ACS) between 2011 and 2017 using targeted maximum likelihood estimation*

|  |  | **Number of study visits (%)** | | | **TMLE estimate** | |
| --- | --- | --- | --- | --- | --- | --- |
|  |  | Total | Anal STI absent | Anal STI present | RR (95% CI) | *p* |
| Age (years) | |  |  |  |  |  |
|  | ≥35 | 4,968 (74.6%) | 4,700 (75.5%) | 268 (61.5%) | Reference |  |
|  | *16-34* | 1,693 (25.4%) | 1,525 (24.5%) | 168 (38.5%) | 1.7 (1.4-2.1) | <0.0001 |
| Education level | |  |  |  |  |  |
|  | *Low (no college/university)* | 1,534 (23.0%) | 1,416 (22.8%) | 118 (27.1%) | Reference |  |
|  | *High (college/university)* | 5,126 (77.0%) | 4,808 (77.3%) | 318 (72.9%) | 0.9 (0.7-1.1) | 0.21 |
| Condomless anal sex with a casual partner | |  |  |  |  |  |
|  | *No* | 4,875 (73.5%) | 4,671 (75.4%) | 204 (47.0%) | Reference |  |
|  | *Yes* | 1,755 (26.5%) | 1,525 (24.6%) | 230 (53.0%) | 2.5 (2.0-3.0) | <0.0001 |
| Condomless anal sex with a steady partner | |  |  |  |  |  |
|  | *No* | 4,069 (61.6%) | 3,776 (61.2%) | 293 (68.0%) | Reference |  |
|  | *Yes* | 2,533 (38.4%) | 2,395 (38.8%) | 138 (32.0%) | 0.8 (0.6-1.0) | 0.01 |
| Chemsex | |  |  |  |  |  |
|  | *No* | 4,831 (74.5%) | 5,418 (89.2%) | 314 (73.7%) | Reference |  |
|  | *Yes* | 1,657 (25.5%) | 655 (10.8%) | 112 (26.3%) | 2.0 (1.6-2.4) | <0.0001 |
| Alcohol during sex | |  |  |  |  |  |
|  | *No* | 2,789 (42.9%) | 2,622 (43.2%) | 167 (39.1%) | Reference |  |
|  | *Yes* | 3,713 (57.1%) | 3,453 (56.8%) | 260 (60.9%) | 0.8 (0.7-1.0) | 0.01 |
| Group sex | |  |  |  |  |  |
|  | *No* | 4,549 (68.6%) | 4,310 (69.5%) | 239 (54.9%) | Reference |  |
|  | *Yes* | 2,087 (31.5%) | 1,891 (30.5%) | 196 (45.1%) | 1.3 (1.1-1.6) | 0.01 |

For number of study visits: data are presented as n (percentages); for TMLE estimate: the target parameter is presented as RR (95% CI). Explanation of data: RR = risk ratio; 95% CI = 95% confidence interval; p = p-value (significance defined at p-value <0.05); TMLE = targeted maximum likelihood estimation. Chemsex is defined as GBL, GHB, mephedrone, methamphetamine, ketamine, amphetamine, cocaine, or XTC use during or prior to sex. Anal STI means diagnosis with anal chlamydia and/or anal gonorrhoea in the 6 months prior to the follow-up visit. All variables refer to the six months prior to the follow-up visits.

**Figure 1.** *Population attributable fractions (PAF) with 95% confidence interval (CI) for determinants of (A) HIV infection, and (B) anal STI (chlamydia or gonorrhoea) among participants of the Amsterdam Cohort Studies (ACS) between 2011 and 2017*

A.

B.

PAFs were calculated from the RR obtained from TLME and presented as percentages with 95%CI, including only determinants of anal STI and HIV that indicated an increased risk (i.e., RR>1). All included variables refer to the six months prior to the follow-up visits. Chemsex is defined as GBL, GHB, mephedrone, methamphetamine, ketamine, amphetamine, cocaine, or XTC use during or prior to sex. Anal STI means diagnosis with anal chlamydia and/or anal gonorrhoea in the 6 months prior to, or at the follow-up visit. Younger age means aged 16-34 versus ages 35 and above. High education level means having a college or university degree and is compared to low education level, meaning no college or university degree.
